# Slit2/Robo1 signaling promotes intestinal tumorigenesis through Src-mediated activation of the Wnt/β-catenin pathway

**DOI:** 10.18632/oncotarget.3060

**Published:** 2014-12-18

**Authors:** Qian-Qian Zhang, Da-lei Zhou, Yan Lei, Li Zheng, Sheng-Xia Chen, Hong-Ju Gou, Qu-Liang Gu, Xiao-Dong He, Tian Lan, Cui-Ling Qi, Jiang-Chao Li, Yan-Qing Ding, Liang Qiao, Li-Jing Wang

**Affiliations:** ^1^ Vascular Biology Research Institute, Guangdong Pharmaceutical University, Guangzhou, China; ^2^ Storr Liver Center, Westmead Millennium Institute for Medical Research, The Western Clinical School of the Faculty of Medicine, The University of Sydney at the Westmead Hospital, Westmead, NSW 2145, Australia; ^3^ Department of Pathology, Nanfang Hospital, Southern Medical University, Guangzhou, China

**Keywords:** Slit2/Robo1 signaling, intestinal tumors, Src, E-cadherin, Wnt/β-catenin

## Abstract

Slit2 is often overexpressed in cancers. Slit2 is a secreted protein that binds to Roundabout (Robo) receptors to regulate cell growth and migration. Here, we employed several complementary mouse models of intestinal cancers, including the *Slit2* transgenic mice, the Apc^Min/+^ spontaneous intestinal adenoma mouse model, and the DMH/DSS-induced colorectal carcinoma model to clarify function of Slit2/Robo1 signaling in intestinal tumorigenesis. We showed that Slit2 and Robo1 are overexpressed in intestinal tumors and may contribute to tumor generation. The Slit2/Robo1 signaling can induce precancerous lesions of the intestine and tumor progression. Ectopic expression of Slit2 activated Slit2/Robo1 signaling and promoted tumorigenesis and tumor growth. This was mediated in part through activation of the Src signaling, which then down-regulated E-cadherin, thereby activating Wnt/β-catenin signaling. Thus, Slit2/Robo1 signaling is oncogenic in intestinal tumorigenesis.

## INTRODUCTION

Recent studies have suggested that the development of a full-blown malignant colorectal tumor is a progressive process involving multiple stages, including the transition of pre-neoplastic cells into aberrant crypt foci followed by the development of adenoma (AD), which then progresses to adenocarcinoma (AC) and metastatic cancer (MC) [1-3]. However, the molecular mechanisms involved in the initiation of colorectal carcinoma are not completely understood.

Slit2 is a secreted protein known to function through the Roundabout (Robo) receptor as a repulsive axon guidance cue. Robo1 and Robo4 are two receptors of the secretory protein Slit2. Robo1 is mainly present in tumor cells, whereas Robo4 is located primarily in endothelial cells of tumor vessels in CRC [4]. It was previously reported that Slit2 and Robo1 are expressed in a large number of malignant solid tumors and the binding of the functional region of Slit2 to Robo1 could initiate a cascade of events leading to altered cell proliferation, migration, apoptosis, and angiogenesis [5-9]. However, the function of Slit2/Robo1 signaling is cell-type dependent. Although the involvement of Slit2/Robo1 signaling in tumor development has been widely implicated, the biological significance and molecular mechanisms of Slit2/Robo1 signaling in the initiation of colorectal carcinoma are largely undetermined.

Wnt signaling mainly branches into two distinct pathways: canonical and non-canonical. In the canonical pathway, activation of Wnt leads to an accumulation of β-catenin in the nucleus. Constitutive activation of the Wnt signaling pathway is believed to be responsible for the initiation of colorectal carcinoma through formation of aberrant crypt foci and small adenomas [1]. Previous studies have reported that activation of the Slit2/Robo1 signaling could activate the Wnt/β-catenin pathway in gastric cancer [10]. This led us to speculate that the Slit2/Robo1 signaling pathway may likely regulate Wnt/β-catenin signaling and thereby promote the initiation of colorectal carcinoma.

Src is a non-receptor tyrosine kinase that is involved in carcinogenesis. Activation of Src signaling through phosphorylation at Tyrosine (416/419) has been found in metastatic CRC tissues [11-13]. Recent reports have also indicated that Src may be a positive regulator of Wnt/β-catenin signaling and it may de-regulate E-cadherin in colon cancer cells [14-16]. The coincidental activation of Slit2/Robo1 signaling and Src in colorectal carcinoma led us to hypothesize that a potential interaction between the Slit2/Robo1 and Src signaling may be an important mechanism involved in the activation of Wnt/β-catenin signaling.

In this study, we used several animal CRC models to define the possible role and the underlying mechanisms of Slit2/Robo1 signaling in intestinal tumorigenesis.

## RESULTS

### Expression of Slit2 and Robo1 in intestinal tumor tissues

**Figure 1:**
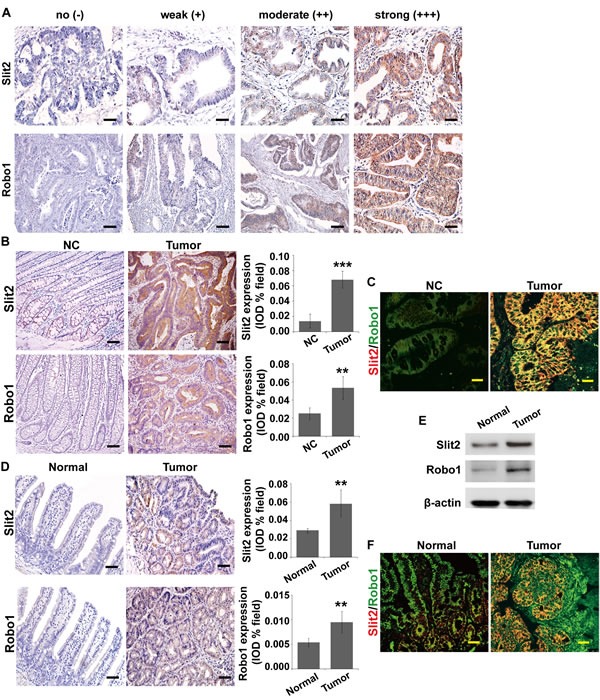
Expression of Slit2 and Robo1 in intestinal tumors Slit2 and Robo1 expression in CRC tissues of various differentiation status was examined by immunohistochemical staining (A). Expression of Slit2 and Robo1 in the matched surrounding non-cancerous colonic tissues (NC) and tumor tissues (Tumor) from stage N0 CRC patients (without metastasis) was examined by IHC (B). Co-localization of Slit2 and Robo1 in the NC and Tumor tissues was examined by immunofluorescent staining (IF) (C). The results are representative of 7 independent specimens. The expression of Slit2 and Robo1 in the normal intestinal mucosa (Normal) and tumor (Tumor) tissues of Apc^Min/+^ mice was examined by IHC staining (D) and Western blotting (E). Co-localization of Slit2 and Robo1 in the normal and tumor tissues of the experimental mice was examined by IF (F). All IF images were obtained under a laser-scanning confocal microscope, and the co-localization of Slit2 and Robo1 was shown by the merged yellow fluorescence (C and F). The results of IHC and IF staining of Apc^Min/+^ mice are representative of 11 mice per group (All mice were 24-week-old). The results of IHC were determined using IPP software, and the quantitative data are expressed as mean ±S.D. *: *P* < 0.05; **: *P* < 0.01; ***: *P* < 0.001. Scale bars: 100 μm (A and B), 25 μm (C and F), and 50 μm (D).

To examine whether the expression and activation of Slit2 and Robo1 are correlated with intestinal tumorigenesis, we first analyzed the protein expression of Slit2 and Robo1 in the tumor tissues relative to their matched surrounding non-cancerous colonic tissues by immunohistochemical analysis. The staining intensity of the cytoplasmic Slit2 and Robo1 in tumor tissues varies from being negligible to strong, as exemplified in Figure 1A. As showed in Table 1, the expression levels of Slit2 and Robo1 in CRC tissues were significantly correlated with lymph node metastasis (*P*=0.001 and *P*<0.001, respectively) and TNM staging (*P*<0.05 and *P*<0.01, respectively). By Spearman rank correlation analysis, the expression level of Slit2 in CRC tissues shows a strong positive correlation with that of Robo1 (*P*<0.001, r=0.459) (Table 2).

**Table 1:** Associations between Slit2 and Robo1 protein expression and various clinicopathological variables in CRC

Clinical Feature	Number	Slit2 expression		P value	Robo1 expression		P value
		Low	High		Low	High	
Gender				0.622			0.244
Male	69	46(66.7%)	23(33.3%)		51(73.9%)	18(26.1%)	
Female	35	25(71.4%)	10(28.6%)		22(62.9%)	13(37.1%)	
Age				0.242			0.321
≤60	56	41(73.2%)	15(26.8%)		37(66.1%)	19(33.9%)	
>60	48	30(62.5%)	18(37.5%)		36(75.0%)	12(25.0%)	
Tumor invasion				0.103			0.094
T_3_	50	38(76.0%)	12(24.0%)		39(78.0%)	11(22.0%)	
T_4_	54	33(61.1%)	21(38.9%)		34(63.0%)	20(37.0%)	
Lymph node Metastasis				0.001			0.012
N_0_	69	55(79.7%)	14(20.3%)		55(79.7%)	14(20.3%)	
N_1_	23	12(52.2%)	11(47.8%)		12(52.2%)	11(47.8%)	
N_2_	12	4(33.3%)	8(66.7%)		6(50.0%)	6(50.0%)	
TNM stage				0.000			0.007
I-II	67	54(80.6%)	13(19.4%)		53(79.1%)	14(20.9%)	
III-IV	37	17(45.9%)	20(54.1%)		20(54.1%)	17(45.9%)	

Pearson χ^2^ test, Low(− and +), High(++ and +++)

We then randomly selected seven pairs of CRC tissues and the matched non-cancerous colonic tissues from the Stage N0 CRC patients (namely, without metastasis) to evaluate the expression profiles of Slit2 and Robo1 by immunohistochemical staining. As exemplified in Figure 1B, CRC tissues express significantly higher amount of Slit2 and Robo1 than the matched non-cancerous colonic tissues. As Slit2 functions through binding to Robo1 in CRC tissues, we made use of two-color immunofluorescent staining to examine if Slit2 and Robo1 co-localize in CRC tissues. As shown in Figure 1C, the CRC tissues exhibit a significantly stronger co-expression of Slit2 and Robo1 than the matched non-cancerous colonic tissues. These results suggest that activation of Slit2/Robo1 signaling may be mechanistically linked to colorectal tumorigenesis.

Apc^Min/+^ mice are a well-established and well-characterized model for the development of spontaneous adenoma in small intestine. This transgenic mouse model has been widely used to facilitate the study for the early initiation of intestinal tumors [17]. We examined the expression profile of Slit2 and Robo1 in the spontaneous adenomatous tissues in Apc^Min/+^ mice by immunohistochemical staining and Western blotting assay. Overexpression of both Slit2 and Robo1 were found in the adenomatous tissues compared with the normal small intestinal tissues (Figure 1, D and E). In addition, a strong co-localization of Slit2 and Robo1 was observed in the adenomatous tumor tissues as opposed to the non-tumorous tissues of the Apc^Min/+^ mice (Figure 1F).

ApcMin/+ mice are less susceptible to develop colon cancer [17]. Thus, in order to examine the expression of Slit2 and Robol in mouse CRC tissues, we made use of the DMH/DSS model in C57BL/6J mice (DMH/DSS-C57) which can readily develop advanced CRC upon exposure to DMH/DSS (Supplementary Figure 1A). More importantly, the pathogenesis of CRC in this model is highly analogous to that observed in human CRC. In our model, a progressive increase in the expression of Slit2 and Robo1, as well as their co-localization was observed during the course of CRC development in DMH/DSS-C57 mice (Supplementary Figure 1, B and D). Moreover, a strong up-regulation of Slit2 and Robo1 was seen in the CRC tissues compared with the non-cancerous tissues of DMH/DSS-C57 mice (Supplementary Figure 1C).

These data strongly suggested that up-regulation and activation of Slit2/Robo1 signaling is likely a significant event associated with intestinal tumorigenesis.

**Table 2:** Correlation analysis of Slit2 and Robo1 expression in CRC

Slit2 expression	Robo1 expression			
	-	+	++	+++
-	9	14	4	0
+	7	30	5	2
++	3	4	10	5
+++	1	5	2	3

r=0.459, P=0.000

### Activation of Slit2/Robo1 signaling induces malignant transformation of the intestinal mucosa *in vivo*

**Figure 2:**
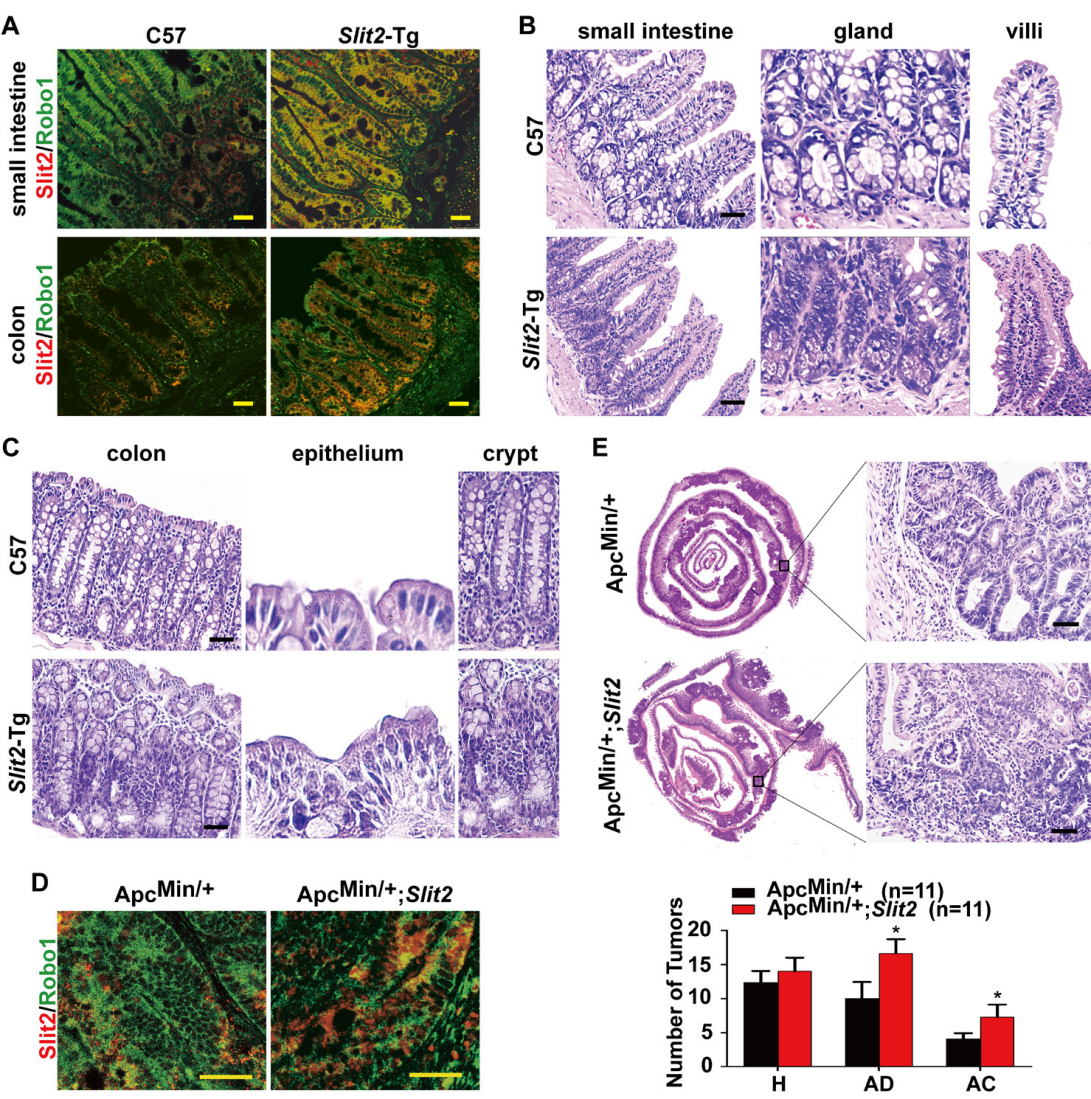
Activation of Slit2/Robo1 signaling accelerates the progression of intestinal tumors Co-localization of Slit2 and Robo1 was detected by IF in small intestine and colon tissues of C57BL/6J (C57) *Slit2*-Tg mice (A). The morphology of the small intestinal mucosa (B) and colonic mucosal (C) tissues from the C57 (12-month-old) and *Slit2*-Tg mice (12-month-old) was examined by H&E staining. Co-localization of Slit2 and Robo1 was detected by IF in the tumor tissues of Apc^Min/+^ and Apc^Min/+^;*Slit2* mice (all in 24-week-old) (D). A holistic view of the histological lesions of intestines was observed on the “whole intestines” under the microscope (E). The number of tumors at each pathological stage, including hyperplasia (H), adenoma (AD) and adenocarcinoma (AC), in each of the Apc^Min/+^ and Apc^Min/+^*Slit2* mouse were quantitatively analyzed, and the data are expressed as mean ± S.D. *: *P* < 0.05. The results (IF and H&E) are representative of 11 mice per group (All mice were 24-week-old). Scale bars: 25 μm (A and D) and 50 μm (B, C and E).

We next investigated the impact of Slit2/Robo1 signaling on the initiation and development of intestinal tumors using the Slit2 transgenic (*Slit2*-Tg) mice model in which Slit2 is genetically overexpressed (Supplementary Table 1 and Supplementary Figure 2, A-C). As shown in Figure 2A, increased co-localization of Slit2 and Robo1 was observed in the small intestinal and colonic tissues of the *Slit2*-Tg mice (12-month-old, female) compared with the intestinal tissues of the age- and gender-matched C57BL/6J mice.

To explore the effect of Slit2/Robo1 signaling activation on the intestinal tumorigenesis, a detailed histological analysis of the intestines of C57BL/6J mice and *Slit2*-Tg mice was performed. As shown in Figure 2B, hyperplasia of the small intestine glands was more frequently seen in the small intestine of *Slit2*-Tg mice than in the C57BL/6J mice, and the villi of *Slit2*-Tg mice were markedly thicker, longer and more fused than those in C57BL/6J mice. Activation of Slit2/Robo1 signaling also affected the histological features of the epithelium of the colon (Figure 2C). In C57BL/6J mice, the crypt surface of the colon only bears a single layer of normal columnar epithelial cells. In contrast, the surface of the colonic crypts in *Slit2*-Tg mice is covered by a large number of hyperplastic epithelial cells. In addition, the colonic crypts of the *Slit2*-Tg mice were more markedly fused compared with those of the C57BL/6J mice.

Furthermore, the Apc^Min/+^ mice were crossed with *Slit2*-Tg mice to generate Apc^Min/+^;*Slit2* mice (Supplementary Table 1 and Supplementary Figure 2, D and E), which were used to evaluate the effect of Slit2/Robo1 signaling activation on the intestinal tumorigenesis. The tumor tissues from the Apc^Min/+^;*Slit2* mice showed an increased co-localization of Slit2/Robo1 compared to the tumor tissues derived from the Apc^Min/+^ mice (Figure 2D). Co-localization of Slit2/Robo1 was also seen in the tumor tissues from the DMH/DSS induced *Slit2*-Tg (DMH/DSS-*Slit2*) mice (Supplementary Figure 3A). On a holistic view of the intestines, large areas of highly condensed nests of neoplastic cells could be identified in the Apc^Min/+^;*Slit2* mice compared to the Apc^Min/+^ mice (Figure 2E). The same results were also seen in the tumor tissues of DMH/DSS-*Slit2* mice (Supplementary Figure 3B).

Together, these results demonstrate that Slit2/Robo1 signaling activation can induce carcinogenesis of intestinal mucosa.

### Activation of Slit2/Robo1 signaling promotes intestinal tumor growth *in vivo*

**Figure 3:**
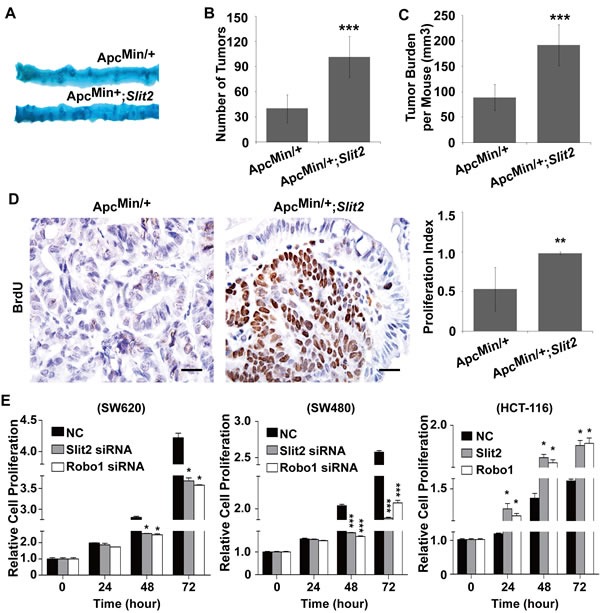
Activation of Slit2/Robo1 signaling promotes the growth of intestinal tumors Following methylene blue staining, the small intestines from the Apc^Min/+^ and Apc^Min/+^;*Slit2* mice were examined macroscopically (A). Overexpression of Slit2 significantly increased the number (B) and size (C) of the tumors. Overexpression of Slit2 resulted in a marked increase in cell proliferation as shown by enhanced BrdU uptake in the tumor tissues of Apc^Min/+^;*Slit2* mice (D). The results of IHC were better presented quantitatively using IPP software (D, right panel). On the other hand, inactivation of the Slit2/Robo1 signaling by siRNA-mediated gene knockdown led to a reduced proliferation of SW620 and SW480 cells (n=6 per group); conversely, overexpression of Slit2 or Robo1 caused an increased proliferation of HCT-116 cells (E). The results of methylene blue staining and IHC staining are representative of 11 mice per group (All mice were 24-week-old). The statistical data were expressed as the mean ±S.D. **: *P* < 0.01, and ***: *P* < 0.001. Scale bars: 20 μm.

Upon macroscopic examination, it was revealed that Apc^Min/+^;*Slit2* mice and the DMH/DSS-*Slit2* mice exhibited a significantly increased tumor incidence (the number of tumors) and tumor burden (average tumor size), compared with Apc^Min/+^ mice and DMH/DSS-C57 mice (Figure 3, A-C, and Supplementary Figure 4, A-C).

Most of the tumors in the experimental mice overexpressing Slit2 were highly proliferative as demonstrated by the significantly increased number of BrdU positive cells(Figure 3D and Supplementary Figure 4D). These data suggested that activation of Slit2/Robo1 signaling promotes the growth of intestinal tumors.

To experimentally confirm the pro-proliferative role of Slit2/Robo1 signaling during the intestinal tumorigenesis, we made use of the siRNA technique to knockdown the expression of Slit2 or Robo1 in SW620 cells and SW480 cells (both cell lines express rich Slit2 and Robo1 [6]). Inactivation of the Slit2/Robo1 signaling led to a significant inhibition of cell proliferation (Figure 3E). Moreover, activation of Slit2/Robo1 signaling could promote the proliferation of HCT-116 cells (this cell line expresses low basal level of Slit2 and Robo1 [6]) (Figure 3E). Thus, Slit2/Robo1 signaling plays a tumor-promoting role during the intestinal tumorigenesis.

### Slit2/Robo1 signaling activates Wnt/β-catenin pathway during intestinal tumorigenesis

**Figure 4:**
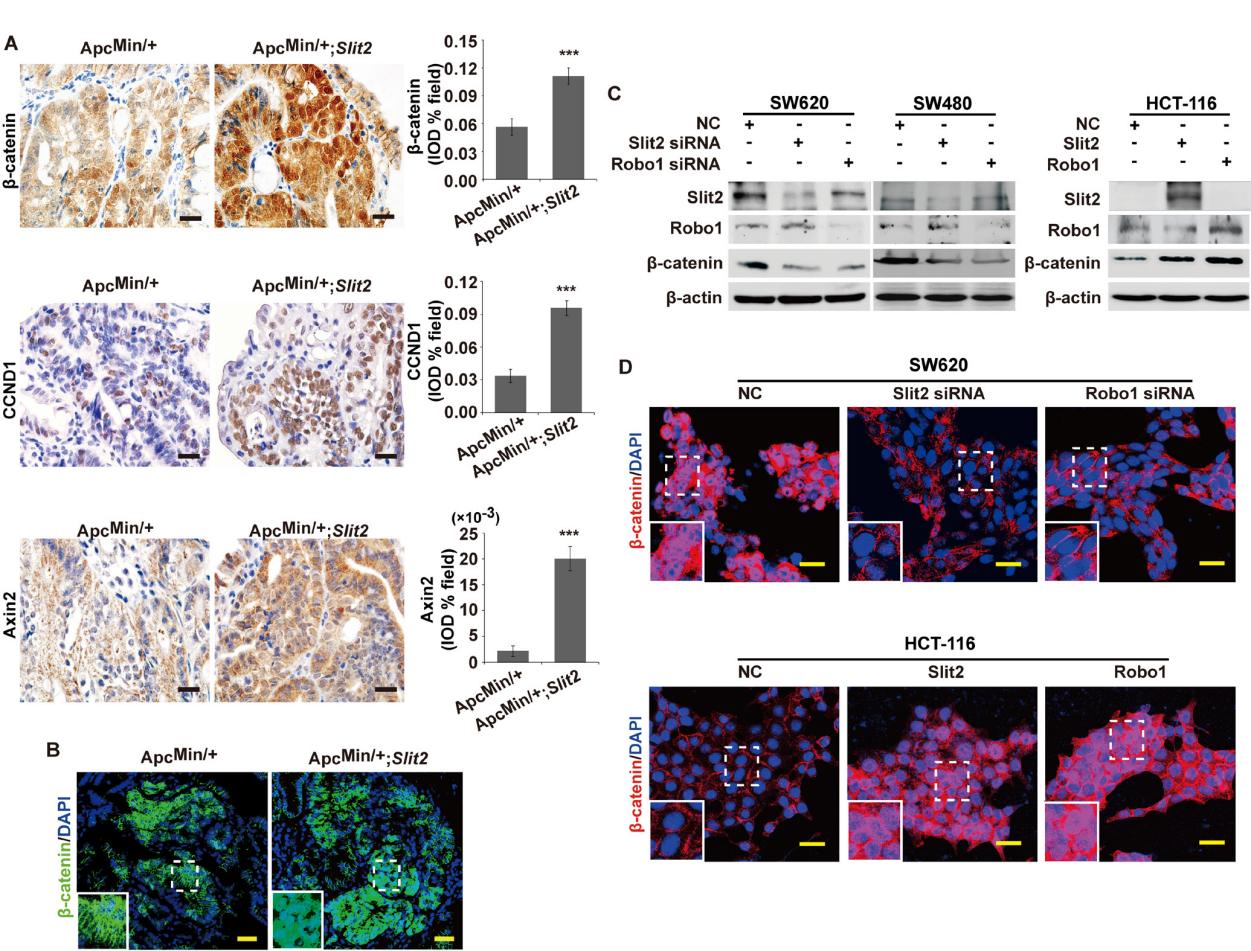
Activation of Slit2/Robo1 signaling led to an activation of Wnt/β-catenin pathway Expression of β-catenin, CCND1 and Axin2 in the tumor tissues of Apc^Min/+^ and Apc^Min/+^;*Slit2* mice was examined by IHC, and the results were quantitatively determined using IPP software and expressed as the mean ± S.D. (A). The subcellular location of β-catenin was examined in the tumor tissues of Apc^Min/+^ and Apc^Min/+^;*Slit2* mice by IF (B). Inactivation of Slit2/Robo1 signaling by suppressing Slit2 or Robo1 expression inhibits the expression of β-catenin in SW620 and SW480 cells, but conversely, activation of Slit2/Robo1 signaling through overexpressing Slit2 or Robo1 expression promotes β-catenin expression in HCT-116 cells (C). Inactivation of Slit2/Robo1 signaling by siRNA against Slit2 or Robo1 suppresses nuclear translocation of β-catenin in SW620 cells, and overexpression of Slit2 or Robo1 promotes nuclear translocation of β-catenin in HCT-116 cells (D). All results represent at least three separate experiments (C and D). The IHC and IF staining of the mouse tissues are representative of 11 mice per group (All mice were 24-week-old). The results of IHC were determined using IPP software, and expressed as the mean ± S.D. *: *P* < 0.05, ***: *P* < 0.001. Scale bars, 20 μm (A) and 25 μm (B and D).

Wnt/β-catenin signaling has been shown to regulate the expression of a subset of genes that promote tumor growth and invasion, and aberrant activation of Wnt/β-catenin signaling has been widely implicated in the development of gastrointestinal cancers [18]. Whether activation of the Slit2/Robo1 signaling facilitates gastrointestinal tumorigenesis by activating the Wnt/β-catenin signaling is undetermined. By immunohistochemistry, we have revealed a significant increase in the expression of β-catenin and two known downstream targets of Wnt/β-catenin pathway cyclin D1 (CCND1) and Axin2 in the tumor tissues of the Apc^Min/+^;*Slit2* and DMH/DSS-*Slit2* mice, as opposed to their respective controls (Figure 4A, and Supplementary Figure 5A). Activation of Wnt/β-catenin signaling results in the nuclear translocation of β-catenin [19]. By immunofluorescence *staining* a strong nuclear translocation of β-catenin has been observed in the tumor cells of the Apc^Min/+^;*Slit2* and DMH/DSS-*Slit2* mice (Figure 4B and Supplementary Figure 5B).

To confirm these *in vivo* findings, we performed *in vitro* studies in SW620 cells, SW480 cells and HCT-116 cells using the siRNA technique and overexpression plasmid to inactivate or activate Slit2/Robo1 signaling, respectively. As shown in Figure 4C, inactivation of Slit2/Robo1 signaling could dramatically suppress the expression of β-catenin in both SW620 and SW480 cells. Further studies by confocal microscopy also confirmed that down-regulation of Slit2 or Robo1 by their respective siRNAs significantly suppressed the nuclear translocation of β-catenin in SW620 cells (Figure 4D). On the other hand, activation of Slit2/Robo1 signaling could promote the expression and the nuclear translocation of β-catenin in HTC-116 cells (Figure 4, C and D). These complementary results suggested that Slit2/Robo1 signaling may initiate the intestinal tumorigenesis by activating the Wnt/β-catenin signaling.

### Slit2/Robo1 signaling activates Wnt/β-catenin signaling and induced epithelial-mesenchymal transition (EMT) through targeting Src-mediated regulation of the E-cadherin pathway

Loss of E-cadherin has been reported to be associated with gastrointestinal tumorigenesis via activating β-catenin signaling [20]. Src has been shown to be one of the key regulators for E-cadherin pathway during tumor growth and metastasis in CRC [16]. It is not clear if the Slit2/Robo1 signaling mediates Wnt/β-catenin pathway through Src/E-cadherin pathway during the intestinal tumorigenesis. To address this question, the expression of pSrc (Tyr 416) was analyzed using immunohistochemical staining. As shown in Supplementary Figure 6A, progressively increased expression of pSrc (Tyr 416) was observed during the development of CRC.

**Figure 5:**
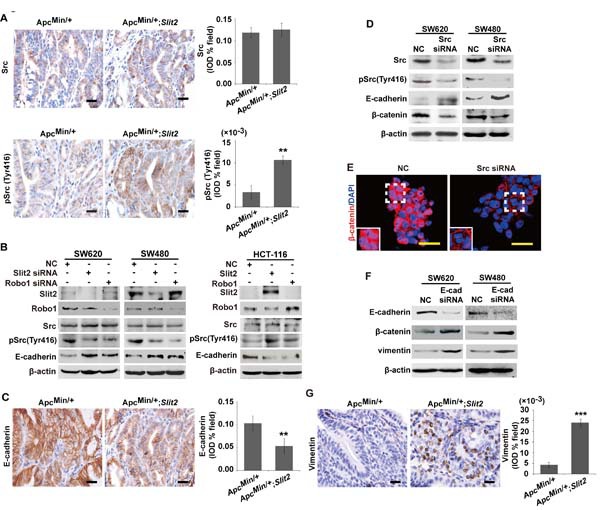
Slit2/Robo1 signaling activates the Src-mediated inhibition of E-cadherin IHC analysis of the expression of total Src and pSrc (Tyr416) in the tumor tissues of Apc^Min/+^ and Apc^Min/+^;*Slit2* mice (A). Inactivation of Slit2/Robo1 significantly reduced the expression of pSrc (Tyr 416) in SW620 and SW480 cells, and activation of Slit2/Robo1 signaling through overexpressing Slit2 or Robo1 expression promotes pSrc (Tyr 416) expression in HCT-116 cells (B). IHC analysis of the expression of E-cadherin in the tumor tissues of the Apc^Min/+^ and Apc^Min/+^;*Slit2* mice (C). Inactivation of Src signaling significantly enhances the expression of E-cadherin but inhibits the expression of β-cateninin in SW620 and SW480 cells (D). Src inactivation in SW620 and SW480 cells also led to a reduced nuclear translocation of β-catenin (E). Inhibition of E-cadherin expression through siRNA technique could promote the expression of β-cateninin and vimentin in SW620 and SW480 cells (F). IHC analysis of the expression of vimentin in the tumor tissues of Apc^Min/+^ and Apc^Min/+^;*Slit2* mice (G). The data in IHC staining are representative of 11 mice per group (All mice were 24-week-old). The results of IHC were determined using IPP software, and the data were expressed as the mean ±S.D. *: *P* < 0.05, **: *P* < 0.01. Scale bars: 20 μm (A, C and G) and 25 μm (E).

Further analysis of the tumor tissues from the Apc^Min/+^;*Slit2* mice and DMH/DSS-*Slit2* mice revealed that activation of Slit2/Robo1 signaling led to an increased pSrc (Tyr 416) in these tumor tissues compared with their respective controls (Figure 5A and Supplementary Figure 6B). To confirm this point, we observed that knockdown of Slit2 and Robo1 by their respective specific siRNAs led to a significant reduction of the pSrc (Tyr 416) in SW620 and SW480 cells (Figure 5B). On the other hand, activation of the Slit2/Robo1 signaling by overexpressing Slit2 or Robo1 could promote the expression of pSrc (Tyr 416) in HTC-116 cells (Figure 5B). In addition, increased Src activity was positively correlated with the enhanced expression of Slit2 or Robo1, suggesting that Src may be a key mediator for the Slit2/Robo1 signaling to regulate Wnt/β-catenin pathway in CRC. However, it was reported that Slit/Robo signaling induces malignant transformation through Hakai1-mediated E-cadherin degradation during the development of CRC [6]. To address if Slit2/Robo1 signaling mediates Src activity via Hakai1, we examined the expression of total Src, pSrc and Hakai1 in SW620 and SW480 cells in which Src or Hakai1 were knocked down. Our results showed that Src and Hakai1 did not exert a mutual or reciprocal regulatory effect (Supplementary Figure 6C). Thus, Slit2/Robo1 signaling may regulate CRC tumorigenesis independent of Src and Hakai1 signaling.

Along with the increased expression of pSrc (Tyr 416) in the intestinal tumor tissues of the Apc^Min/+^;*Slit2* mice and DMH/DSS-*Slit2* mice, activation of Slit2/Robo1 signaling was also found to be associated with a marked decrease in the expression of E-cadherin in the tumor tissues (Figure 5C and Supplementary Figure 6D). In addition, inhibition of Src pathway by siRNA technique markedly enhanced the expression of E-cadherin in SW620 and SW480 cells (Figure 5D), which was associated with a decreased expression and nuclear translocation of β-catenin in these cells (Figure 5, D and E). Our results showed that inactivation of the Slit2/Robo1 signaling led to a significant up-regulation of E-cadherin and inhibition of β-catenin expression. In order to test if E-cadherin has a direct regulatory role for β-catenin, we performed a siRNA-mediated knockdown of E-cadherin in SW620 and SW480 cells. As shown in Figure 5F, inhibition of E-cadherin expression could promote β-catenin expression. These data show that Slit2/Robo1 signaling may induce Wnt signaling via down-regulation E-cadherin.

It was reported that Slit2/Robo1 signaling can induces EMT in CRC cells. Therefore, we examined the expression of vimentin (an EMT marker) in E-cadherin down-regulated cells and intestinal tumor tissues by Western blotting. As shown in Figure 5, F and G and Supplementary Figure 6E, down-regulation of E-Cadherin or activation of Slit2/Robo1 signaling could promote the expression of vimentin.

Taken together, our data demonstrated that Slit2/Robo1 signaling can activate Wnt/β-catenin signaling and induce EMT through Src-mediated down-regulation of the E-cadherin pathway.

## DISCUSSION

**Figure 6:**
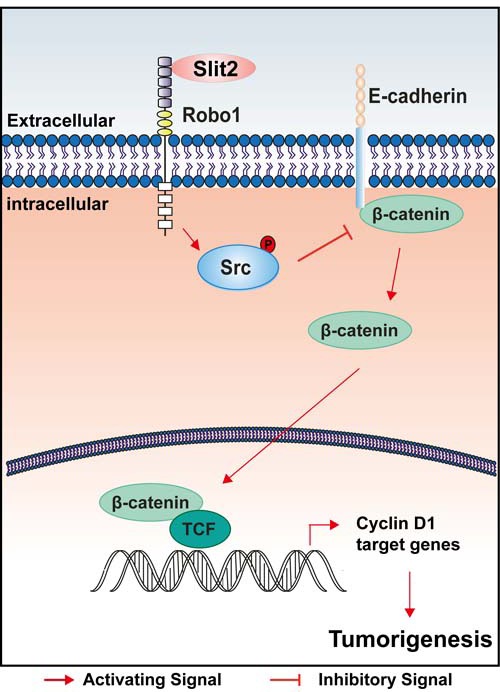
A schematic illustration of how activation of Slit2/Robo1 signaling might promote early initiation of intestinal tumorigenesis

This study has clearly demonstrated an oncogenic role of Slit2/Robo1 signaling during the tumorigenesis and progression of intestinal cancer. Our findings support a model that up-regulation of Slit2 directly results in the recruitment of the adaptor protein Robo1, which then phosphorylates Src and suppresses the expression of E-cadherin, leading to an increased activity of Wnt/β-catenin signaling, and thereby promotes the tumor growth and accelerates the progression of the intestinal tumors (Figure 6).

The expression profile of Slit2 shows no clear trend among different cancers. Down-regulation of Slit2 has been reported in several other cancers, including those from lung, breast, cervix, skin and ovary [8, 21-23]. In CRC, Slit2 has been shown to induce apoptosis, inhibit cell migration and thus was believed to be a tumor suppressor [24, 25]. Although, our *in vitro* data in one of the cell lines (SW480) were not consistent with what has been previously published [24], we have consistently shown in two CRC cell lines (SW480 and SW620) and by a complementary approach in another cell line HCT-116, that activation of Slit2/Robo1 signaling may exert oncogenic effects during the CRC development. To support our data, it has been previously published that increased expression of Slit2 was present in other malignant tumors, including malignant melanoma, mucinous adenocarcinoma of the rectum, invasive carcinoma of breast, squamous carcinoma of stomach, hepatocellular carcinoma, and metastatic CRC [6, 7]. Apparently, the role of Slit2/Robo1 signaling in human CRC is still a matter of debate. It has been well established that activation of the Apc/β-catenin pathway and CpG island hypermethylation are two distinct mechanisms contributing to the development of CRC. It was reported that Slit2 gene was silenced by hypermethylation of its promoter region in CRC patients [24, 25]. However, previous studies have reported overexpression of Slit2 and Robo1 in CRC tissues as well as a progressive increase of Slit2 in the colonic tissues during the development of CRC [6, 7]. The data generated in our current study suggest not only support the published data, but also indicate that increased Slit2/Robo1 signaling in the human CRC tissues, cell lines, and mouse CRC models is likely related to the activation of the Apc/β-catenin pathway.

In this study, we employed two complementary mouse models of intestinal cancers: a genetically engineered spontaneous small intestinal adenoma mouse model and a carcinogen-induced colon carcinoma model to analyze the expression and function of Slit2 in the development of intestinal cancers. These two models can mimic the process of *in vivo* intestinal tumorigenesis, and allow us to successfully define the expression and function of Slit2/Robo1 signaling during the tumorigenesis and progression of the intestinal cancers. We have demonstrated that Slit2 was overexpressed in the early-stage intestinal tumors with its expression progressively increased during the development of intestinal cancer. Clearly, up-regulation of Slit2 is a significant event associated with intestinal tumorigenesis.

Previous reports have indicated a clear association between the Slit2 expression and tumor invasion in several cancer types, including CRC, glioma, and skin cancer [6-8, 26, 27]. Our study has confirmed that increased Slit2/Robo1 signaling may exert an oncogenic role during the early initiation of intestinal tumorigenesis. In our studies, hyperplastic crypts and epithelium, fused villi and crypts were frequently seen in the small intestine and colon of the 12-month-old *Slit2*-Tg mice compared with the age- and gender-matched C57BL/6J mice. As reported previously, the villi of the 8-week-old *Slit2*-Tg mice were noticeably thicker, longer, enlarged and outnumbered compared with those of the age- and gender-matched wild-type mice [28]. These data are consistent with the previously reports that the earliest identifiable pre-neoplastic biomarkers for human and rodent models of colon cancer are epithelial cell deregulation and the appearance of aberrant crypt foci [29-31]. These morphologically altered intestinal structures are benign at the early stage but if persist for long enough, cancers are more likely to derive. The fact that activation of Slit2/Robo1 signaling led to an altered epithelial structure in our study further demonstrates that Slit2 can drive the precancerous lesions of the intestine to intestinal cancers.

The mechanisms by which Slit2 promotes intestinal tumorigenesis and cancer progression are unclear. Previous studies have demonstrated that Slit2 can regulate EMT and induces malignant transformation of the colonic epithelial cells through Hakai-mediated E-cadherin degradation [6, 7]. Meanwhile, activation of the Src protein kinase has also been reported to be an early event in colonic carcinogenesis [32]. Src activation is a multifactorial process involving an interaction between Src and many other receptor tyrosine kinases, such as EGFR, PDGFR, FGFR, CSF-1R, HER2/neu and c-Met [33-39]. In the current work, we have provided compelling evidence that an increased expression and activity of the Slit2/Robo1 signaling can activate the Src tyrosine kinases, resulting in a cascade of events leading to the activation of Wnt/β-catenin signaling. E-cadherin, an important member of the extracellular matrix network controlling cellular integrity and stability, is likely an important intermediator linking Slit2/Robo1 signaling and activation of Src and subsequently Wnt/β-catenin signaling. Our work demonstrated that Src- and Hakai1-mediated E-cadherin down-regulation are two independent regulatory mechanisms of Slit2/Robo1 signaling in CRC tumorigenesis. However, more intensive studies are warranted to elucidate the direct interaction between the Slit2/Robo1 signaling and Src protein kinase during the intestinal tumorigenesis.

In summary, our data have demonstrated that activation of Slit2/Robo1 signaling is critically involved in the intestinal tumorigenesis and Slit2 may constitute a potential biomarker for the early detection and therapy of intestinal cancers.

## MATERIALS AND METHODS

### Reagents and antibodies

1, 2-Dimethylhydrazine (DMH) and dextran sodium sulfate (DSS) were purchased from Sigma-Aldrich (St. Louis, Missouri, USA). The siRNA duplex for Slit2 was obtained from Santa Cruz Biotechnology Inc. (Santa Cruz, California, USA), and the siRNA duplexes for Robo1, Src and E-cadherin were obtained from Guangzhou RiboBio Co., Ltd. (Guangzhou, China). The Slit2 overexpression plasmid was obtained from ViGene Biosciences Inc. (Rockville MD, USA). Robo1 overexpression plasmid was donated by Dr. Xuesong Yang (Jinan University, China). BrdU and mouse anti-BrdU antibody (B2531, diluted at 1:1000) were obtained from Sigma-Aldrich (St. Louis, Missouri, USA). The following primary antibodies were used for immunohistochemical (IHC), immunofluorescence (IF) analysis and Western blotting (WB) assays: rabbit anti-Slit2 (ab7665) and rabbit anti-Robo1 (ab7279) (both diluted at 1:100 for IHC/IF and 1:2000 for WB) were obtained from Abcam (Cambridge, UK); rabbit anti-Src (PB0080, diluted at 1:100 for IHC and 1:400 for WB) was obtained from Boster (Wuhan, China); rabbit anti-pY416Src (#6943s, diluted at 1:100 for IHC, and 1:1000 for WB) and rabbit anti-β-actin (#4967, diluted at 1:1000) were obtained from Cell Signaling Technology, Inc. (Danvers, Massachusetts, USA); mouse anti-E-cadherin (#610181, diluted at 1:100 for IHC and 1:5000 for WB) and mouse anti-β-catenin (#610154, diluted at 1:100 for IHC, and 1:2000 for WB) were purchased from BD Transduction Laboratories (Franklin Lakes, NJ); rabbit anti-Axin2 (AB21962a, diluted at 1:100) was purchased from Sangon (Shanghai, China); and rabbit anti-Hakai1 (bs-8386R, diluted at 1:500) was purchased from Bioss (Beijing, China).

### Patients and tissue samples

A total of 104 cases of colonic tumor tissues and their matched non-tumorous colonic tissues as well as the corresponding clinical data were collected from the Department of Pathology, Nanfang Hospital. The 104 patients consist of 69 men and 35 women, with a median age of 59.5 years (ranging from 22 to 86 years). Pathological classification of the tumor staging (pTNM) was based on the guidelines of International Union Against Cancer (UICC). Written informed consent from each patient was obtained prior to the initiation of this study. Pathologic diagnosis was performed by two independently pathologists. The study has been approved by the Institutional Ethics Committee of the Nanfang Hospital and the Guangdong Pharmaceutical University.

### Animals and treatment

The C57BL/6J mice were obtained from the Guangdong Medical Laboratory Animal Center. The Apc^Min/+^ mice were obtained from the Jackson Laboratory (Bar. Harbor, Maine, USA). The Slit2 transgenic mice were generated and donated by Dr. Jianguo Geng (University of Michigan, USA) according to the published procedures [40], and more detailed description of the experimental procedures is provided as the Supplementary Materials and Methods. Verification of the successful transgene expression is provided as the Supplementary Figures 2 A-C. Use of the animals in this project was approved by the Ethics Committee of the Center of Laboratory Animals, Guangdong Pharmaceutical University. All mice were maintained under a 12-h light/dark cycle in a climate-controlled room at 24 ± 2°C and 60 ± 5% humidity. All surgical procedures were performed under diethylether anesthesia. BrdU was intraperitoneally injected into the mice at a dose of 0.1 mg/g body weight 1.5 hours before sacrifice.

### Cell culture and transfection

Colorectal carcinoma cell lines SW620, SW480 and HCT-116 were obtained from the cell bank of the Chinese Academy of Sciences (Shanghai, China). Cells were incubated at 37°C in a humidified chamber containing 5% CO_2_ and maintained in Dulbecco's Modified Eagle's Medium (DMEM, GIBCO) with 10% fetal bovine serum (FBS), 100 U/mL penicillin, and 100 μg/mL streptomycin. Lipofectamine 2000 (Invitrogen) was used to transfect siRNAs (final concentration 100 nM) (to SW620 and SW480 cells) or Slit2 and Robo1 overexpression plasmids (to HCT-116 cells).

### Analysis of cell proliferation

SW620, SW480 and HCT-116 cells were plated at density of 10,000, 2,500 and 2,500 cells/well, respectively. Then transfected with siRNAs (to SW620 and SW480 cells) or overexpression plasmids (to HTC-116 cells) in each of the 96-well plate. Cell viability was evaluated using the standard the MTT assay. The data were obtained from three independent experiments each assayed in quadruplicate.

### Analysis of intestinal tumors

Following sacrifice, the intestines were removed and sliced longitudinally, rinsed with saline, fixed with Notox Histo Fixative for overnight, stained with 0.1% methylene blue, and spread onto microscope slides. Each small intestine was divided lengthwise into three equal sections: proximal, middle, and distal segments. The number of tumors was counted, the tumor length (L) and width (W) were measured with a digital caliper under the dissecting microscope and the volume (V) was calculated with the formula (L×W2)×0.5236. In order to holistically assess and compare the tumor incidence between each group, all separate intestinal sections from each animal were pinned together to anatomically “restore” the entire intestinal structure. The number of the macroscopic tumors in the “restored” intestines was counted. The “restored” intestines were then embedded in paraffin blocks, cut into sections of 4 μm thickness, and stained with standard H&E procedures. The intestinal tumors and malignant foci were further assessed under the microscope.

### Histological and immunoblotting analyses

The intestinal tissues were fixed in 10% neutral formalin solution for 24 hours, embedded in paraffin, and cut into sections of 4 μm thickness. For routine histology, the sections were stained with hematoxylin & eosin (H&E). For immunohistochemistry and immunofluorescent assays, the sections were deparaffinized, dehydrated with graded alcohol, and blocked with 10% bovine serum albumin at 37°C for 30 min. The sections were then incubated with relevant primary antibodies for overnight at 4°C, washed three times in PBS, and then incubated with horseradish peroxidase (HRP)-conjugated secondary antibodies or fluorescently labeled secondary antibodies for 1 hour at room temperature. The sections were treated with diaminobenzidine solution to develop color and then counterstained with hematoxylin before being viewed under microscope.

Following sacrifice, the intestines were removed and sliced longitudinally, rinsed with saline, and observed under the dissecting microscope. The macroscopic tumors were cut off from the intestines (tumor tissues) and the parts of intestinal tissues that were not affected by tumors were cut off as the normal mucosal tissues. The total proteins from the tissues and cells were prepared using RIPA buffer, and immunoblotting assays were performed as previously described [41].

### Statistical analysis

The data are presented as the mean ± standard deviation (SD). Unless otherwise stated, the differences between groups were analyzed using a Student*'*s *t-*test when only two groups were compared. All tests were two-sided. The Pearson Chi-square test (χ^2^) or Fisher's exact test for proportion was used to analyze the relationship between protein expression and clinicopathologic characteristics. Spearman rank correlation coefficient test was used to analyze the correlation between Slit2 and Robo1. Difference was considered statistically significant if a *P* value is < 0.05. The protein expression level in the immunohistochemical slides were determined by measuring the cumulative integrated optical density (IOD) using IPP software.

## SUPPLEMENTARY MATERIAL, FIGURES, TABLES



## References

[1] Sancho E, Batlle E, Clevers H (2004). Signaling pathways in intestinal development and cancer. Annu Rev Cell Dev Biol.

[2] Ansher AF, Lewis JH, Fleischer DE, Cattau EL, Collen MJ, O'Kieffe DA, Korman LY, Benjamin SB (1989). Hyperplastic colonic polyps as a marker for adenomatous colonic polyps. Am J Gastroenterol.

[3] Terzic J, Grivennikov S, Karin E, Karin M (2010). Inflammation and colon cancer. Gastroenterology.

[4] Grone J, Doebler O, Loddenkemper C, Hotz B, Buhr HJ, Bhargava S (2006). Robo1/Robo4: differential expression of angiogenic markers in colorectal cancer. Oncol Rep.

[5] Latil A, Chene L, Cochant-Priollet B, Mangin P, Fournier G, Berthon P, Cussenot O (2003). Quantification of expression of netrins, slits and their receptors in human prostate tumors. Int J Cancer.

[6] Zhou WJ, Geng ZH, Chi S, Zhang W, Niu XF, Lan SJ, Ma L, Yang X, Wang LJ, Ding YQ, Geng JG (2011). Slit-Robo signaling induces malignant transformation through Hakai-mediated E-cadherin degradation during colorectal epithelial cell carcinogenesis. Cell research.

[7] Wang B, Xiao Y, Ding BB, Zhang N, Yuan X, Gui L, Qian KX, Duan S, Chen Z, Rao Y, Geng JG (2003). Induction of tumor angiogenesis by Slit-Robo signaling and inhibition of cancer growth by blocking Robo activity. Cancer Cell.

[8] Kim HK, Zhang H, Li H, Wu TT, Swisher S, He D, Wu L, Xu J, Elmets CA, Athar M, Xu XC, Xu H (2008). Slit2 inhibits growth and metastasis of fibrosarcoma and squamous cell carcinoma. Neoplasia.

[9] Dunaway CM, Hwang Y, Lindsley CW, Cook RS, Wu JY, Boothby M, Chen J, Brantley-Sieders DM (2011). Cooperative signaling between Slit2 and Ephrin-A1 regulates a balance between angiogenesis and angiostasis. Molecular and cellular biology.

[10] Shi R, Liu W, Liu B, Xu Z, Chen L, Zhang Z (2013). Slit2 expression and its correlation with subcellular localization of beta-catenin in gastric cancer. Oncol Rep.

[11] Kopetz S (2007). Targeting SRC and epidermal growth factor receptor in colorectal cancer: rationale and progress into the clinic. Gastrointest Cancer Res.

[12] Bolen JB, Veillette A, Schwartz AM, Deseau V, Rosen N (1987). Analysis of pp60c-src in human colon carcinoma and normal human colon mucosal cells. Oncogene Res.

[13] DeSeau V, Rosen N, Bolen JB (1987). Analysis of pp60c-src tyrosine kinase activity and phosphotyrosyl phosphatase activity in human colon carcinoma and normal human colon mucosal cells. J Cell Biochem.

[14] Kajla S, Mondol AS, Nagasawa A, Zhang Y, Kato M, Matsuno K, Yabe-Nishimura C, Kamata T (2012). A crucial role for Nox 1 in redox-dependent regulation of Wnt-beta-catenin signaling. FASEB J.

[15] Yokoyama N, Malbon CC (2009). Dishevelled-2 docks and activates Src in a Wnt-dependent manner. J Cell Sci.

[16] Avizienyte E, Wyke AW, Jones RJ, McLean GW, Westhoff MA, Brunton VG, Frame MC (2002). Src-induced de-regulation of E-cadherin in colon cancer cells requires integrin signalling. Nature cell biology.

[17] Rosenberg DW, Giardina C, Tanaka T (2009). Mouse models for the study of colon carcinogenesis. Carcinogenesis.

[18] Ou CY, LaBonte MJ, Manegold PC, So AY, Ianculescu I, Gerke DS, Yamamoto KR, Ladner RD, Kahn M, Kim JH, Stallcup MR (2011). A coactivator role of CARM1 in the dysregulation of beta-catenin activity in colorectal cancer cell growth and gene expression. Mol Cancer Res.

[19] Martinez-Moreno JM, Munoz-Castaneda JR, Herencia C, Oca AM, Estepa JC, Canalejo R, Rodriguez-Ortiz ME, Perez-Martinez P, Aguilera-Tejero E, Canalejo A, Rodriguez M, Almaden Y (2012). In vascular smooth muscle cells paricalcitol prevents phosphate-induced Wnt/beta-catenin activation. Am J Physiol Renal Physiol.

[20] Orsulic S, Huber O, Aberle H, Arnold S, Kemler R (1999). E-cadherin binding prevents beta-catenin nuclear localization and beta-catenin/LEF-1-mediated transactivation. J Cell Sci.

[21] Singh RK, Indra D, Mitra S, Mondal RK, Basu PS, Roy A, Roychowdhury S, Panda CK (2007). Deletions in chromosome 4 differentially associated with the development of cervical cancer: evidence of slit2 as a candidate tumor suppressor gene. Human genetics.

[22] Narayan G, Goparaju C, Arias-Pulido H, Kaufmann AM, Schneider A, Durst M, Mansukhani M, Pothuri B, Murty VV (2006). Promoter hypermethylation-mediated inactivation of multiple Slit-Robo pathway genes in cervical cancer progression. Molecular cancer.

[23] Dallol A, Da Silva NF, Viacava P, Minna JD, Bieche I, Maher ER, Latif F (2002). SLIT2, a human homologue of the Drosophila Slit2 gene, has tumor suppressor activity and is frequently inactivated in lung and breast cancers. Cancer research.

[24] Chen WF, Gao WD, Li QL, Zhou PH, Xu MD, Yao LQ (2013). SLIT2 inhibits cell migration in colorectal cancer through the AKT-GSK3beta signaling pathway. Int J Colorectal Dis.

[25] Dallol A, Morton D, Maher ER, Latif F (2003). SLIT2 axon guidance molecule is frequently inactivated in colorectal cancer and suppresses growth of colorectal carcinoma cells. Cancer research.

[26] Yiin JJ, Hu B, Jarzynka MJ, Feng H, Liu KW, Wu JY, Ma HI, Cheng SY (2009). Slit2 inhibits glioma cell invasion in the brain by suppression of Cdc42 activity. Neuro Oncol.

[27] Qi C, Lan H, Ye J, Li W, Wei P, Yang Y, Guo S, Lan T, Li J, Zhang Q, He X, Wang L (2014). Slit2 promotes tumor growth and invasion in chemically induced skin carcinogenesis. Lab Invest.

[28] Zhou WJ, Geng ZH, Spence JR, Geng JG (2013). Induction of intestinal stem cells by R-spondin 1 and Slit2 augments chemoradioprotection. Nature.

[29] Roncucci L, Medline A, Bruce WR (1991). Classification of aberrant crypt foci and microadenomas in human colon. Cancer Epidemiol Biomarkers Prev.

[30] Nascimbeni R, Donato F, Ghirardi M, Mariani P, Villanacci V, Salerni B (2002). Constipation, anthranoid laxatives, melanosis coli, and colon cancer: a risk assessment using aberrant crypt foci. Cancer Epidemiol Biomarkers Prev.

[31] Zoghbi S, Drouin E, Claustre J, Bara J, Scoazec JY, Plaisancie P (2007). Intestinal MUC2 and gastric M1/MUC5AC in preneoplastic lesions induced by 1,2-dimethylhydrazine in rat: a sequential analysis. Int J Oncol.

[32] Cartwright CA, Meisler AI, Eckhart W (1990). Activation of the pp60c-src protein kinase is an early event in colonic carcinogenesis. Proc Natl Acad Sci U S A.

[33] Levitzki A (1996). SRC as a target for anti-cancer drugs. Anticancer Drug Des.

[34] Irby R, Mao W, Coppola D, Jove R, Gamero A, Cuthbertson D, Fujita DJ, Yeatman TJ (1997). Overexpression of normal c-Src in poorly metastatic human colon cancer cells enhances primary tumor growth but not metastatic potential. Cell Growth Differ.

[35] Mao W, Irby R, Coppola D, Fu L, Wloch M, Turner J, Yu H, Garcia R, Jove R, Yeatman TJ (1997). Activation of c-Src by receptor tyrosine kinases in human colon cancer cells with high metastatic potential. Oncogene.

[36] Courtneidge SA, Fumagalli S, Koegl M, Superti-Furga G, Twamley-Stein GM (1993). The Src family of protein tyrosine kinases: regulation and functions. Dev Suppl.

[37] LaVallee TM, Prudovsky IA, McMahon GA, Hu X, Maciag T (1998). Activation of the MAP kinase pathway by FGF-1 correlates with cell proliferation induction while activation of the Src pathway correlates with migration. J Cell Biol.

[38] Luttrell DK, Lee A, Lansing TJ, Crosby RM, Jung KD, Willard D, Luther M, Rodriguez M, Berman J, Gilmer TM (1994). Involvement of pp60c-src with two major signaling pathways in human breast cancer. Proc Natl Acad Sci U S A.

[39] Rahimi N, Hung W, Tremblay E, Saulnier R, Elliott B (1998). c-Src kinase activity is required for hepatocyte growth factor-induced motility and anchorage-independent growth of mammary carcinoma cells. J Biol Chem.

[40] Yang XM, Han HX, Sui F, Dai YM, Chen M, Geng JG (2010). Slit-Robo signaling mediates lymphangiogenesis and promotes tumor lymphatic metastasis. Biochem Biophys Res Commun.

[41] Xu H, He JH, Xiao ZD, Zhang QQ, Chen YQ, Zhou H, Qu LH (2010). Liver-Enriched Transcription Factors Regulate MicroRNA-122 That Targets CUTL1 During Liver Development. Hepatology.

