# Supplementary material for: Slit2/Robo1 signaling promotes intestinal tumorigenesis through Src-mediated activation of the Wnt/β-catenin pathway
Source: Oncotarget. 2014 Dec 18;6(5):3123–35. doi: 10.18632/oncotarget.3060 (PMC4413642; doi:10.18632/oncotarget.3060)
Supplement: Supplementary file 1 [file oncotarget-06-3123-s001.pdf]

Slit2/Robo1 signaling promotes intestinal tumorigenesis through Src-mediated activation of the Wnt/β-catenin pathway

Supplementary Material

Generation of the Slit2 transgenic mice

The full-length human Slit2 cDNA was cloned into pCEP4F vector with a DNA sequence coding for a Flag fragment driven by CMV promoter and injected into the pronuclei of fertilized C57×CBA F1 oocytes. The chimeric mice were backcrossed at least 6 times to C57 mice to generated *Slit2*-Tg mice with C57BL/6 background. Founder mice were confirmed to exhibit stable transmission of the transgene to their progeny. Mice were genotyped from genomic DNA isolated from toe biopsies by PCR (the primers were shown in Supplementary Table 1). The Slit2 transgenic mice were viable and fertile. The expression of Slit2 in the intestine of *Slit2*-Tg mice was verified by IHC and Western blotting.

HYPERLINK "http://mc.manuscriptcentral.com:80/LongRequest/jpath?DOWNLOAD=TRUE&PARAMS=xik_e7Et238Ed3N3cXnGo6Hr23cwA9muTXwBBXPc5TUJV4aZgLYrF5ikp3AWgHba7AZKDttbYwAoE3JCJi3yWZK5xe8mYbJz7vuwT14kaGtMdZKLtahX8EUDgket9TQ7NQKinD1YGAJVTojJvGFnUoy1qmLP8Q9jAsXoFatcKgJN8XDw1KSQjSUZuZ1Ja7mE5MUn8xCZ4CwJ6xpv38B8rkXPyntT8D" \t "_new"

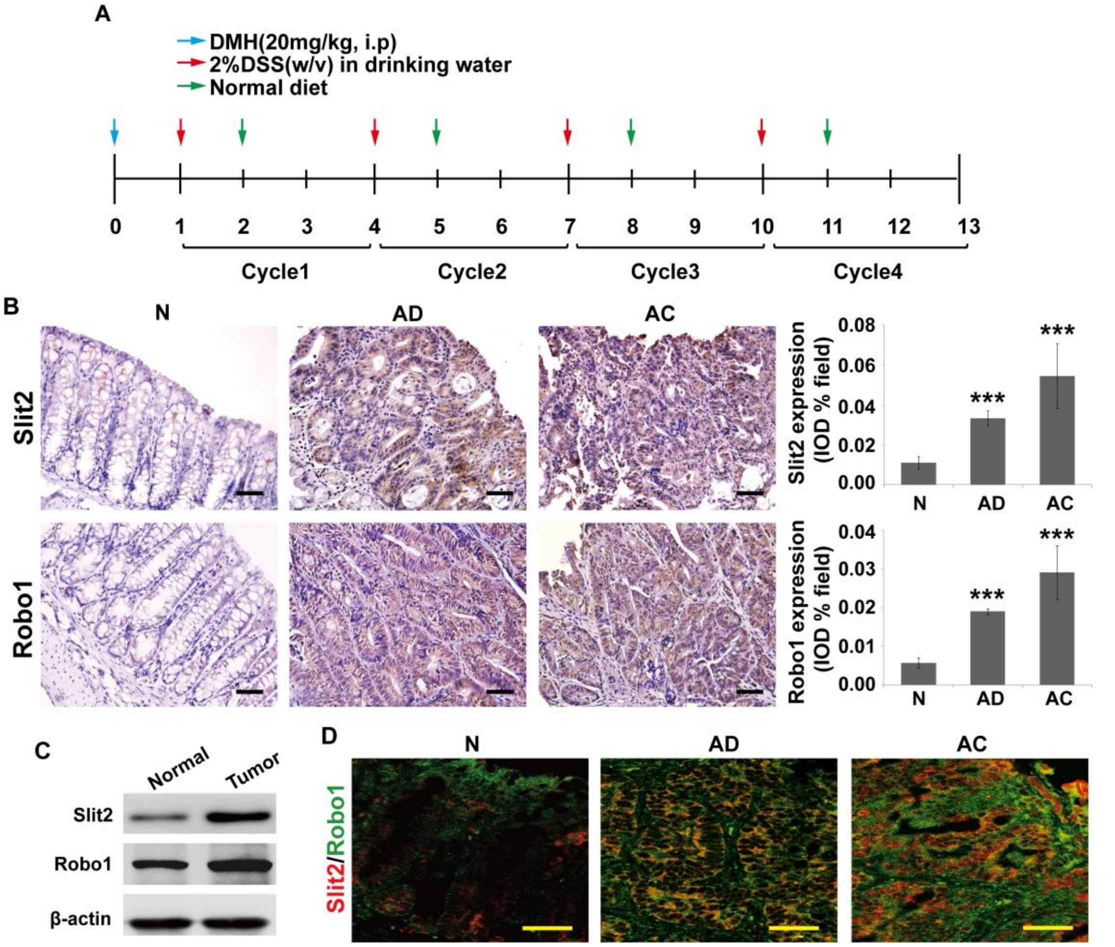

Supplementary Figure 1: Expression of Slit2 and Robo1 in colonic tumors. Schematic illustration of generating DMH/DSS-induced colonic cancer in mice (**A**). The dynamic changes of the expression of Slit2 and Robo1 during the development of DMH/DSS-induced colon cancer was examined by IHC staining (**B**), and better expressed quantitatively (B, right panel). N: normal mucosa; AD: adenoma; and AC: adenocarcinoma. The expression of Slit2 and Robo1 in the normal colonic mucosa (Normal) and the tumor tissues (Tumor) of DMH/DSS-induced CRC model were also detected by Western blotting (**C**). Co-localization of Slit2 and Robo1 in the colonic tissues during the development of the DMH/DSS-induced CRC was examined by IF (**D**). The results of IHC were determined using IPP software, and the quantitative data

are expressed as mean ± S.D, *: $P < 0.05$; **: $P < 0.01$; ***: $P < 0.001$. Scale bars: 50 μm (**B**) and 25 μm (**D**).

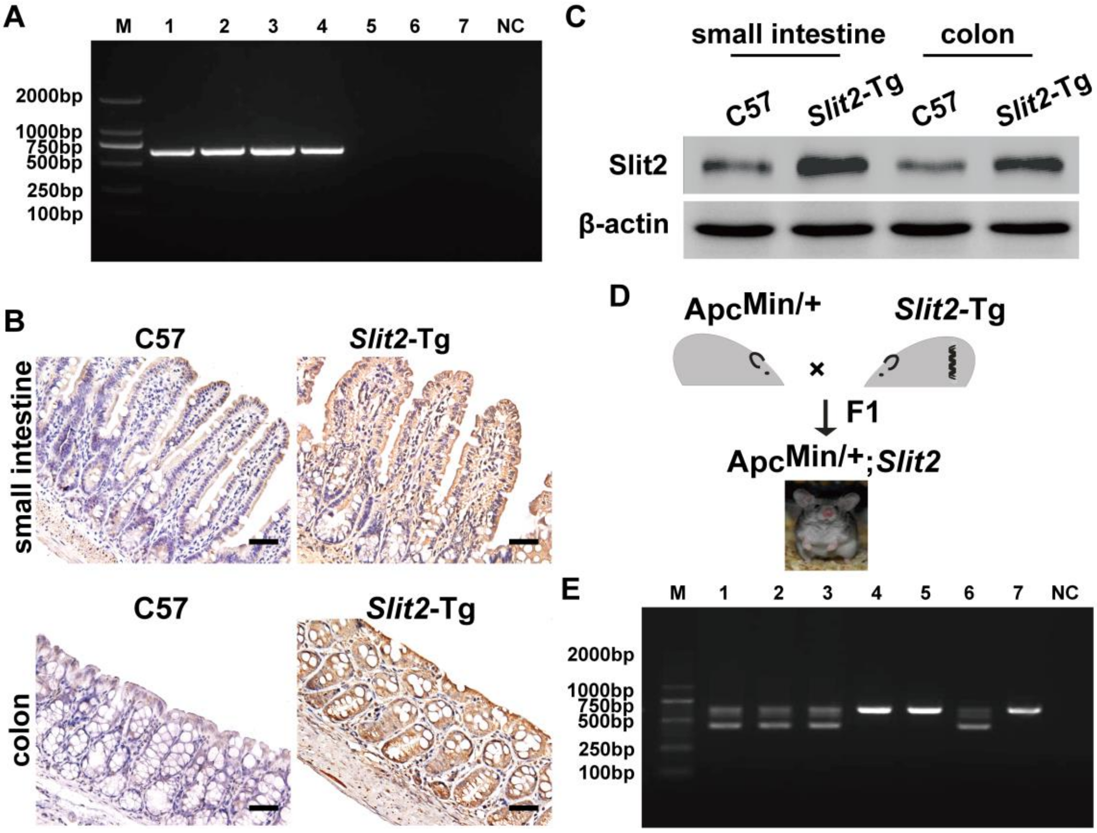

Supplementary Figure 2: Construction of Slit2-overexpressing mice. Genomic DNA samples isolated from mouse tails were subjected to genotyping by PCR using specific primers for *Slit2*. Panel A. M: molecular weight marker D2000; NC: negative control; Lanes 1-4: Slit2 overexpressing mice; Lanes 5-7: C57 mice. Overexpression of Slit2 was verified using IHC staining (**B**) and Western blotting (**C**) in small intestines and colons of C57 and *Slit2*-Tg mice. A schematic diagram of generating Apc$^{Min/+}$;*Slit2* F1 experimental mice carrying both the Slit2 gene and Apc mutant allele is shown in Panel **D**. PCR screening of Apc$^{Min/+}$;*Slit2* mice using primers that detect both wild-type and mutant alleles of *Apc* is shown in Panel **E**. M: molecular

weight marker D2000; NC: negative control; Lanes 1-3 and 6: Apc$^{Min/+}$;*Slit2* mice (*Apc* wild-type and mutant alleles, 619 bp and 331 bp); Lanes 4, 5 and 7: Slit2 mice (*Apc* wild-type allele, 619 bp) (**E**).

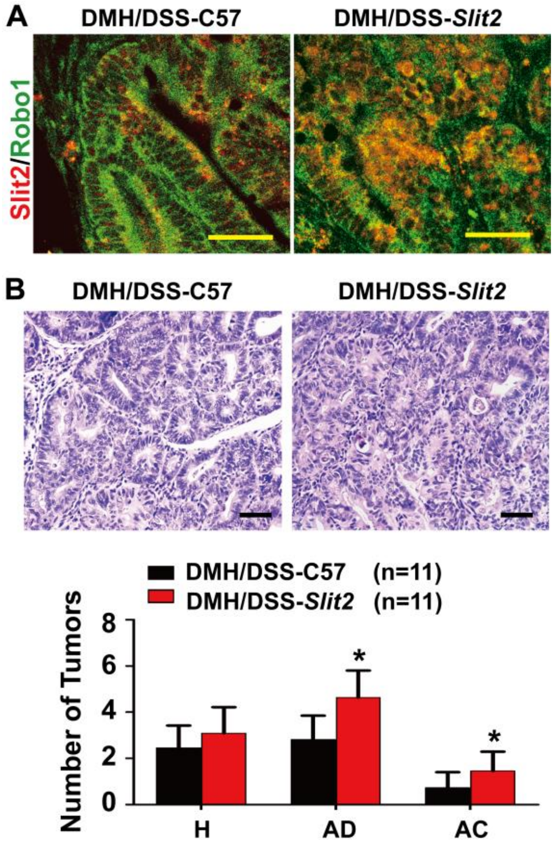

Supplementary Figure 3: Activation of Slit2/Robo1 signaling accelerates the progression of colonic tumors. Co-localization of Slit2 and Robo1 was detected by IF in the tumor tissues of DMH/DSS-C57 and DMH/DSS-*Slit2* mice (all in 24-week-old) (**A**). The number of tumors at each pathological stages, including hyperplasia (H), adenoma (AD) and adenocarcinoma (AC), in each of the DMH/DSS-C57 and DMH/DSS-*Slit2* mice were quantitatively analyzed, and the data are expressed as mean ± S.D, *: $P < 0.05$ (**B**). The results (IF and H&E) are representative of 11 mice per group (All mice were 24-week-old). Scale bars: 25 μm (**A**) and 50 μm (**B**).

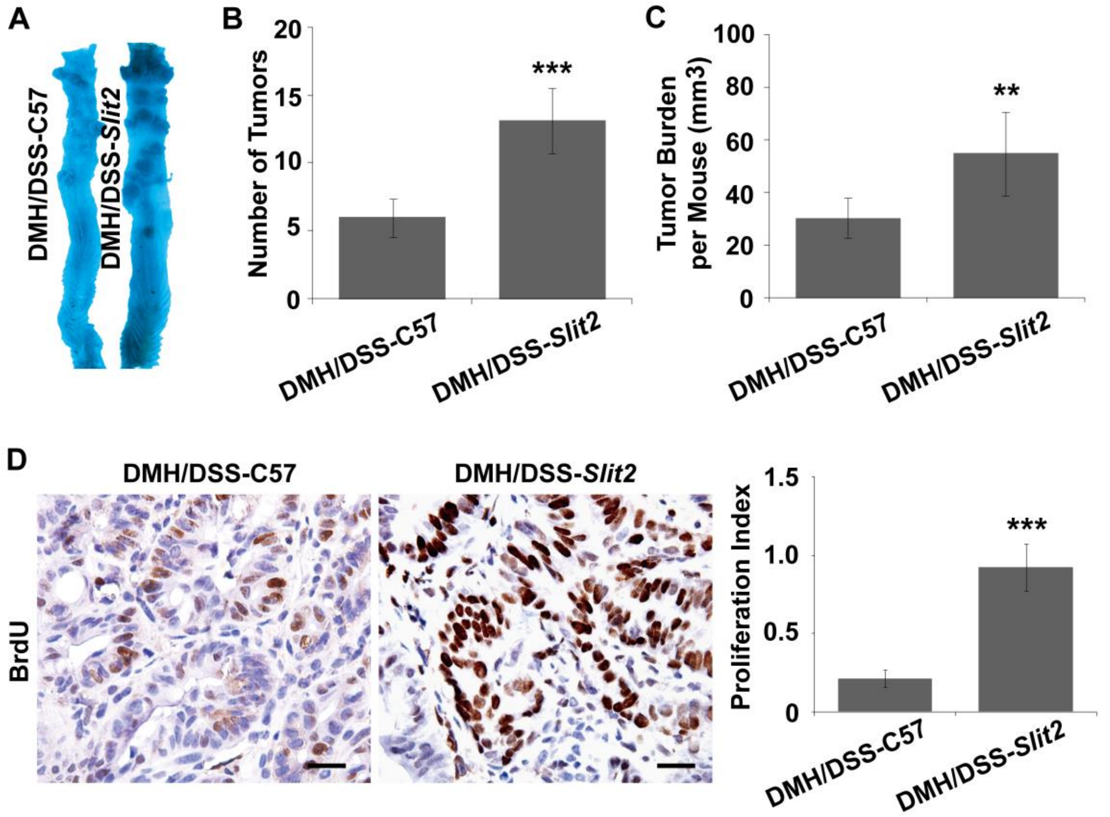

Supplementary Figure 4: Activation of Slit2/Robo1 signaling promotes the growth of colonic tumors. Following methylene blue staining, the colons of DMH/DSS-C57 and DMH/DSS-Slit2 mice were examined macroscopic (**A**). Overexpression of Slit2 significantly increased the number (**B**) and size (**C**) of tumors. Overexpression of Slit2 also resulted in a marked increase in cell proliferation as shown by enhanced BrdU uptake in the tumor tissues of DMH/DSS-*Slit2* mice (**D**). The results of IHC staining were quantitatively determined using IPP software (**D, right panel**). The results of methylene blue staining and IHC staining are representative of 11 mice per group (All mice were 24-week-old). Data were expressed as the mean ± S.D, **: $P < 0.01$, and ***: $P < 0.001$. Scale bars: 20 μm.

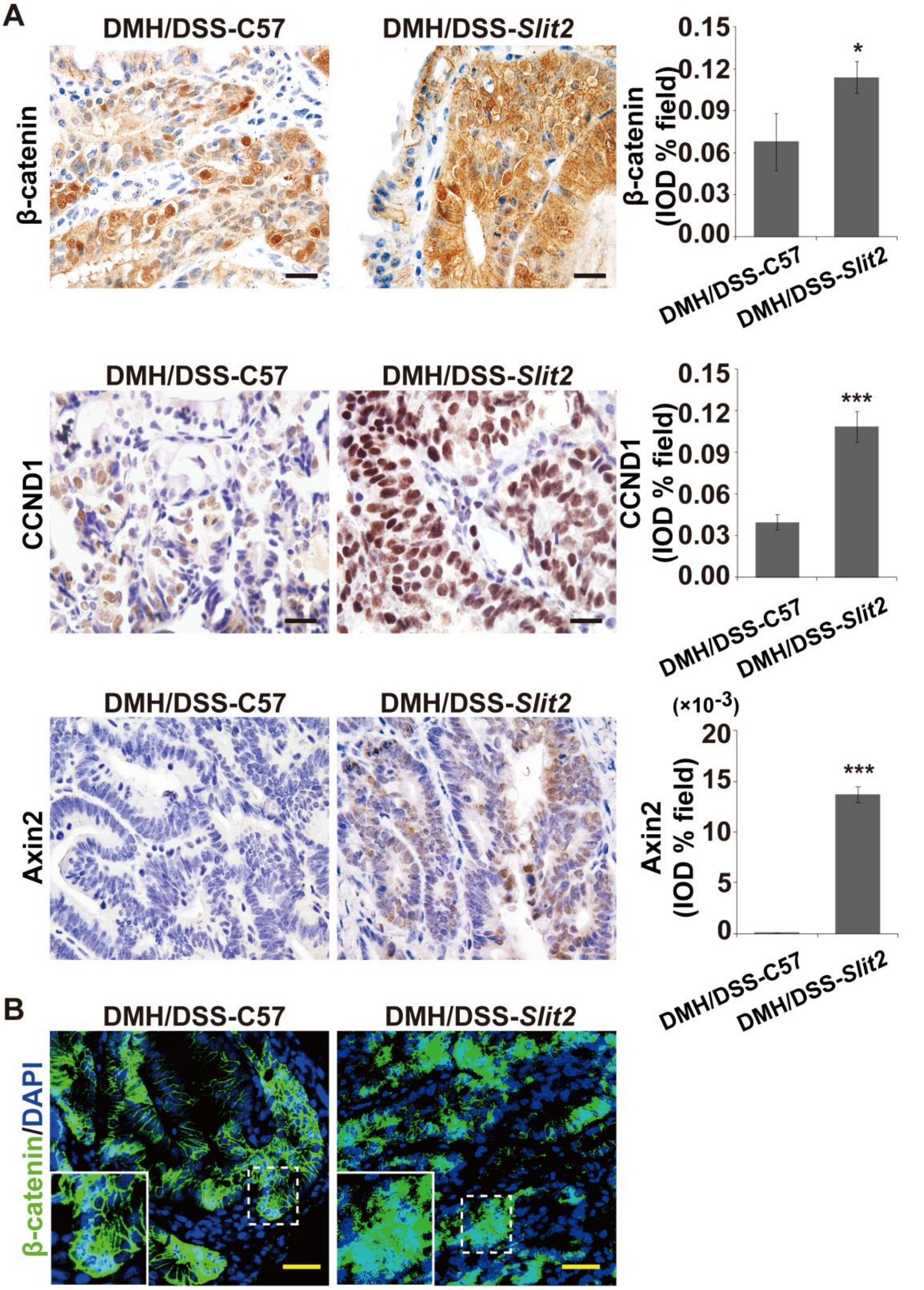

Supplementary Figure 5: Activation of Slit2/Robo1 signaling led to an activation of Wnt/β-catenin pathway. Expression of β-catenin and the target genes of Wnt/β-catenin pathway (CCND1 and Axin2) in the tumor tissues of DMH/DSS-C57 and

DMH/DSS-*Slit2* mice were examined by IHC, and the quantitative results were determined using IPP software and expressed as the mean ± S.D. (**A**). The subcellular localization of β-catenin was examined in the tumor tissues of DMH/DSS-C57 and DMH/DSS-*Slit2* mice by IF (**B**). The IHC and IF staining of the mouse tissue specimens are representatives of 11 mice per group (All mice were 24-week-old). The quantitative results of IHC were determined using IPP software, and expressed as the mean ± S.D, ***: $P < 0.001$. Scale bars, 20 μm (**A** and **B**) and 25 μm (**C**).

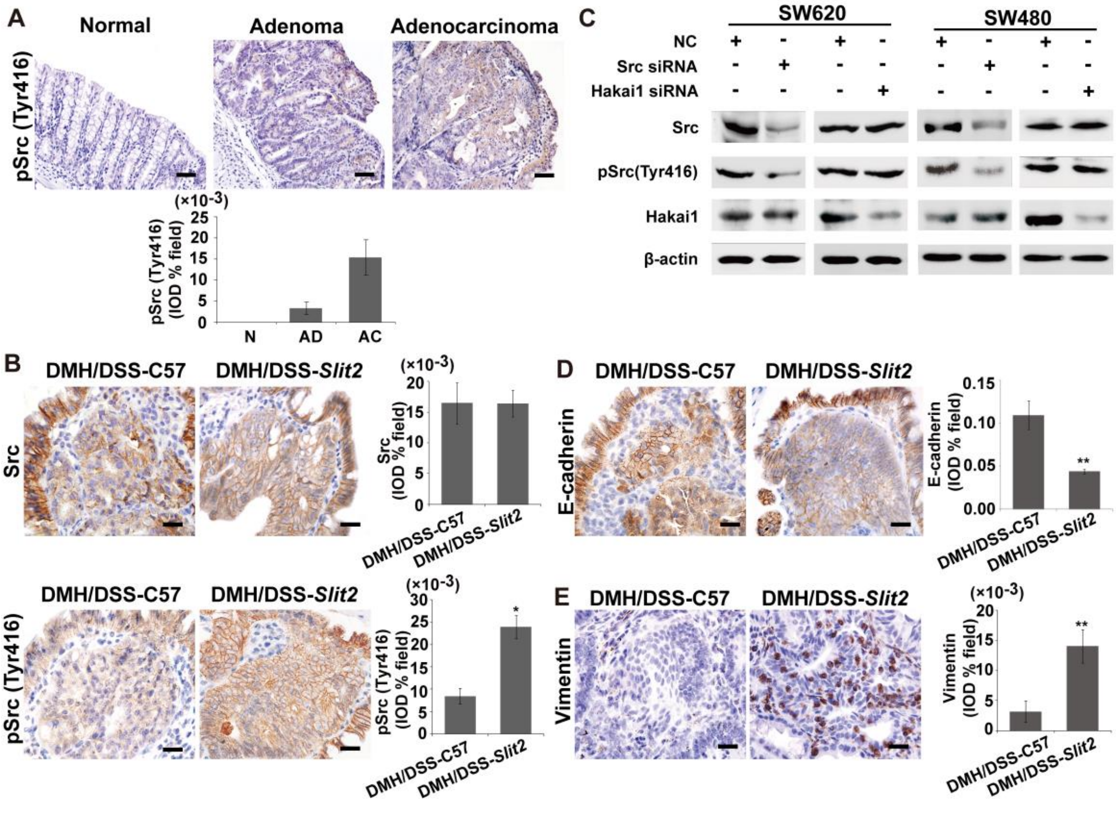

Supplementary Figure 6: Slit2/Robo1 signaling regulates Src-mediated inhibition of E-cadherin. Expression of pSrc (Tyr 416) in the colonic tissues during the stage-wise development of the DMH/DSS-induced CRC in mice was examined by IHC (**A**),

and quantitatively presented (**A, bottom panel**). N: normal mucosa; AD: adenoma; AC: adenocarcinoma. IHC staining analysis the Src and pSrc (Tyr416) expression in the tumor tissues of DMH/DSS-C57 and DMH/DSS-*Slit2* mice (**B**). Western blotting analysis of the expression of Src, pSrc (Tyr 416) and Hakai1 by suppressing Src or Hakai1 expression in SW620 and SW480 cells, respectively (**C**). IHC staining analysis of the expression of E-cadherin (**D**) and Vimentin (**E**) in the tumor tissues of DMH/DSS-C57 and DMH/DSS-*Slit2* mice. The IHC staining photos are representative of 11 mice per group (All mice were 24-week-old). The results of IHC were determined using IPP software, and expressed as the mean ± S.D, *: $P < 0.05$, **: $P < 0.01$. Scale bars, 50 µm (**A**) and 20 µm (**B, D** and **E**).

Supplementary Table 1. Primers for PCR genotyping of Slit2-overexpressing Mice

**Primer**	**Sequence (5'→3')**
Slit2-F	CCCTCCGGATCCTTTACCTGTCAAGGTCCT
Slit2-R	TGGAGAGAGCTCACAGAACAAGCCACTGTA
F1- *Apc* wild-type typing	GCC ATC CCT TCA CGT TAG
F2- *Apc* min typing	TTC TGA GAA AGA CAG AAG TTA
R- *Apc* universal typing	TTC CAC TTT GGC ATA AGG C

Primers F1/R combination generates an amplicon of 619 bp wild-type (WT) allele sequence and primers F2/R combination generates one amplicon of 331 bp mutant (Mut) allele sequence.
